# Older Nonmedical and Medical Cannabis Users: Health Characteristics, Cannabis Use Patterns, and Cannabis Sources

**DOI:** 10.1093/geroni/igab046.3051

**Published:** 2021-12-17

**Authors:** Namkee Choi, Diana DiNitto

**Affiliations:** 1 University of Texas, Austin, Texas, United States; 2 University of Texas at Austin, Austin, Texas, United States

## Abstract

Despite rapidly growing number of older medical cannabis users, research on them is scant. In this study, we examined medical and nonmedical cannabis users aged 50+ on health-related characteristics and cannabis use patterns and sources. Hypotheses were that compared to nonmedical users, medical users are more likely to have physical and mental health problems, use healthcare services, discuss their drug use with a healthcare professional, use cannabis more frequently, and purchase cannabis from a medical dispensary and other sources rather than obtain it as a gift, share someone else’s, or use other means. We used the 2018 and 2019 National Survey on Drug Use and Health data (N=17,685 aged 50+; male=8,030; female=9,655) and multivariable logistic regression analysis to test hypotheses. Of the sample, 8.9% reported past-year cannabis use. Of past-year users, 18.5% reported any medical use. Of medical users, 70.9% reported exclusive medical use and 29.1% reported using medically and nonmedically. A large proportion obtained cannabis from private/informal sources. Any medical use, compared to nonmedical use, was associated with lower odds of alcohol use disorder but higher odds of discussing drug use with a healthcare professional (AOR=4.18, 95% CI=2.53-6.89), more days of use (AOR=2.56, 95% CI=1.35-4.86 for 200-365 days), and purchase at a medical cannabis dispensary (AOR=4.38, 95% CI=2.47-7.76). Medical and nonmedical users did not differ on physical health, and both had high behavioral health problem rates. However, only a small portion discussed their drug use with a healthcare professional. More healthcare professional attention to older cannabis users is needed.

